# Clinical Audit of the Emergency Management of Convulsive Status Epilepticus in Children Subsequently Admitted to Paediatric Intensive Care

**DOI:** 10.7759/cureus.110398

**Published:** 2026-06-07

**Authors:** David Thomas, William P Whitehouse

**Affiliations:** 1 Paediatric Intensive Care, Nottingham University Hospitals NHS Trust, Nottingham, GBR; 2 Paediatric Neurology, Nottingham University Hospitals NHS Trust, Nottingham, GBR; 3 School of Medicine, University of Nottingham, Nottingham, GBR

**Keywords:** buccal midazolam, children, clinical audit, convulsive status epilepticus, guideline adherence, out of hospital treatment, pediatric emergency department (ped), rectal diazepam, refractory status epilepticus, service evaluation and improvement

## Abstract

Objective

The objective of this study was to evaluate the emergency pre-paediatric intensive care unit (PICU) management of children with convulsive status epilepticus subsequently admitted to PICU, to identify opportunities for improving early management.

Methods

Retrospective case note review of children admitted to one PICU over two years (2004-2005) with a diagnosis of status epilepticus. We compared the pre-PICU management with national guidelines.

Results

Thirty-six of 739 (5%) of all PICU admissions were for epileptic seizures. Of the 32 children beyond the neonatal period, 26 had notes available for audit. Prolonged febrile seizure was the most common aetiology (9/26; 34%). Of the 19 children transferred by ambulance, six (32%) received pre-hospital treatment. Only one of these six received an appropriate dose of rectal diazepam. None received midazolam pre-hospital. The mean time from seizure onset to time of first emergency drug dose in those without pre-hospital treatment was 81 (range 30-265) minutes. Pre-PICU, in the Emergency Department, the Advanced Paediatric Life Support guidelines for status epilepticus were strictly followed in 10/26 (38%) while 7/26 (27%) received more than two doses of benzodiazepines in total. Of the 36 benzodiazepine doses given, nine (25%) were inappropriately low. The sequence of drugs suggested in the national guideline was not followed in 9/26 (35%), and 21 (81%) were intubated and ventilated.

Conclusion

As delay in giving emergency drugs is avoidable, we recommend that paramedical staff give pre-hospital emergency drugs more readily, and in the recommended dose. The use of buccal or intramuscular midazolam by paramedics and ambulance crews would facilitate prompt pre-hospital treatment. We also recommend continuous education and training of hospital staff about the importance of generally following guidelines, especially on the doses of emergency drugs.

## Introduction

Convulsive status epilepticus (CSE) is the most common neurological emergency in childhood associated with significant morbidity and mortality [1-8]. It is an important cause of admission to paediatric intensive care units (PICUs) [1,4,9]. Aicardi and Chevrie in 1970 found a mortality rate of 11% [10], and more recent studies report mortality rates of 3-8% [1,2,4-7]. Although the outcome of CSE is mainly determined by its cause [1-8], the duration of the seizure is also important [4-8]. In addition, the longer the duration of the seizure, the more difficult it may be to terminate [8-12].

The children admitted to PICUs represent the minority of those more severely affected. For example, about a third of all children receiving emergency department (ED) treatment for epileptic seizures, including status epilepticus, were subsequently admitted to PICU in a clinical audit in the UK in 2010-2011 [13]. Only about 10% of the children treated in the ED for epileptic seizures required anaesthesia to control their refractory status epilepticus, consistent with other institutions.

Strategies for managing CSE in children have evolved over time and depend on the setting, whether in the community pre-hospital [11,14], in the emergency department [12,15], or in the PICU [16,17]. A recent systematic review found intramuscular midazolam slightly quicker than buccal or nasal midazolam and was effective in a slightly higher proportion of children with CSE. These three routes were superior to rectal diazepam [18]. Benzodiazepines have been the mainstay of first-line treatment for over 40 years, although respiratory depression can be significant [14,19,20]. Rectal diazepam or buccal midazolam remain in general use for those without intravenous access [15,16,21-26], although intramuscular midazolam may be a better choice [18]. 

In this institutional audit, we aimed to identify avoidable factors that may contribute to the need for PICU admission.

This article was previously presented as an abstract at the annual Royal College of Paediatrics and Child Health (RCPCH) meeting in York, United Kingdom, March 2007 [27].

## Materials and methods

This was a retrospective clinical audit of unselected consecutive children admitted to the PICU with a documented clinical diagnosis of status epilepticus from January 2004 to December 2005. The PICU was in a regional paediatric intensive care and paediatric neurosciences centre, Queen's Medical Centre, Nottingham University Hospitals NHS Trust, Nottingham, United Kingdom, serving a total population of 1.2 million (200,000 children and young people), admitting approximately 600 children a year. Patients aged 29 days to 15 years inclusive were included in the audit, and those with non-convulsive status epilepticus were excluded.

This was a non-interventional, purely observational, retrospective clinical audit of the process and outcome of the pre-PICU admission management of CSE in children. As it was part of the routine quality assurance and quality improvement process, and not clinical research, research ethics approval was not required. It was a registered clinical audit.

Data extraction

We searched the PICU admission database of doctors' and nurses' medical notes and discharge summaries of admissions for that period. All potential cases were identified using the terms “status”, “epilepticus”, “seizure”, “convulsion”, “fit” and “respiratory depression”. A standard data collection form was used to collect demographic and medical history data, and history of the hospital episode leading to the PICU admission, including contemporaneous notes made in the Paediatric Emergency Department and hospital wards where relevant.

Analyses

Audit of Outcome

Mann-Whitney U test was carried out to investigate the relation between the aetiology of CSE and the duration of PICU stay, and the relation between pre-hospital management with seizure duration and PICU stay. Pearson product-moment correlation coefficient (r) was used to examine the relation between the time from seizure onset to first antiseizure medication (ASM) and seizure duration and duration of PICU stay.

Audit of Process

**Figure 1:**
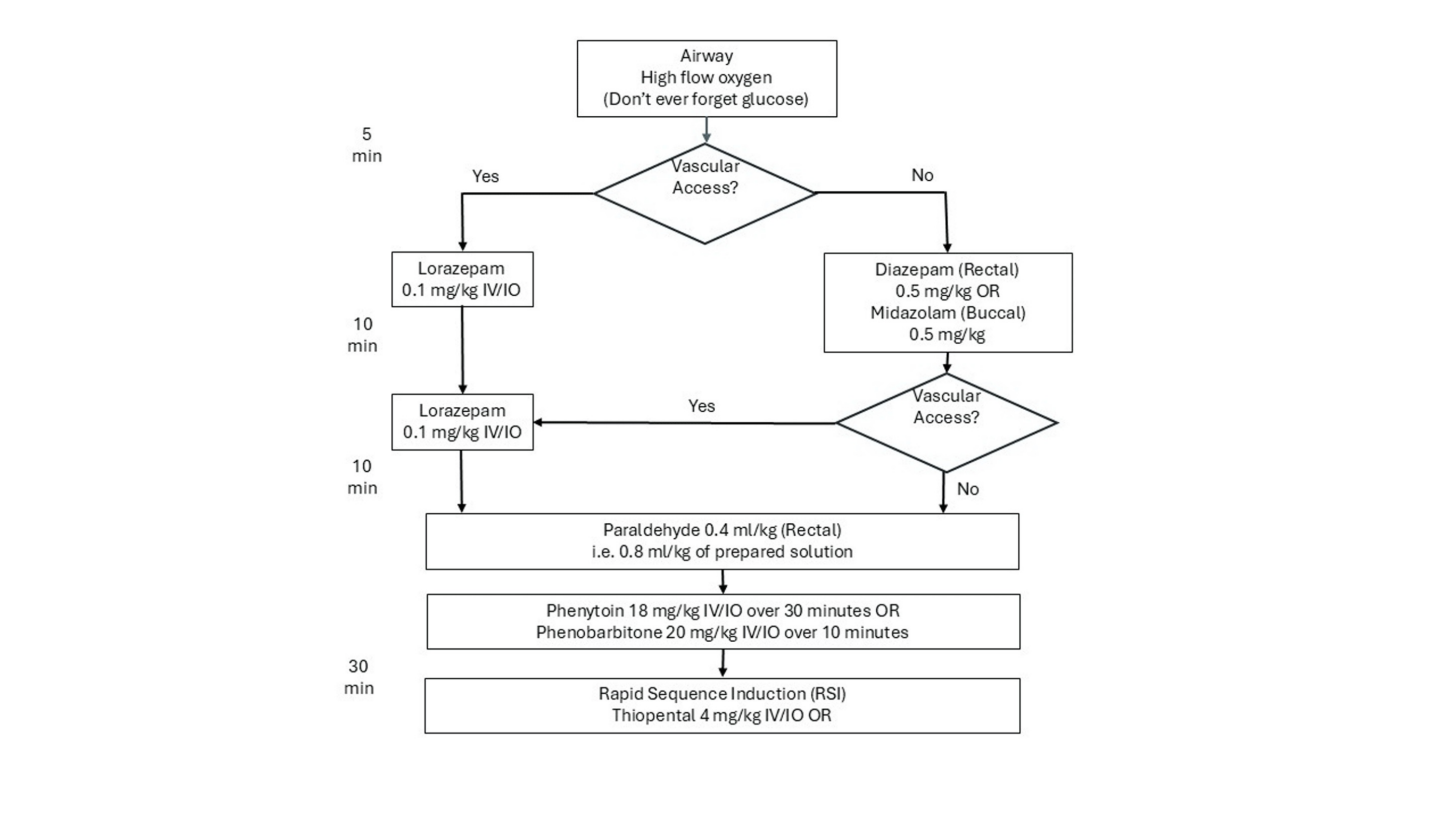
Summary flow chart of the abridged APLS guidelines, 4th edition, 2004 APLS: Advanced Paediatric Life Support [21], IV/IO: intravenous/intraosseous

The hospital's status epilepticus guideline for children, as used in the Paediatric Emergency Department and children's wards, followed the Advanced Paediatric Life Support (APLS), 4th edition [21]. All resident and consultant paediatricians in the United Kingdom are expected to have had APLS training and regular updates. Summary flow charts were displayed in the resuscitation rooms (Figure 1). Based on the APLS guidelines, a dose of less than 80% of the recommended dose was considered too low and greater than 120% too high.

**Figure 2:**
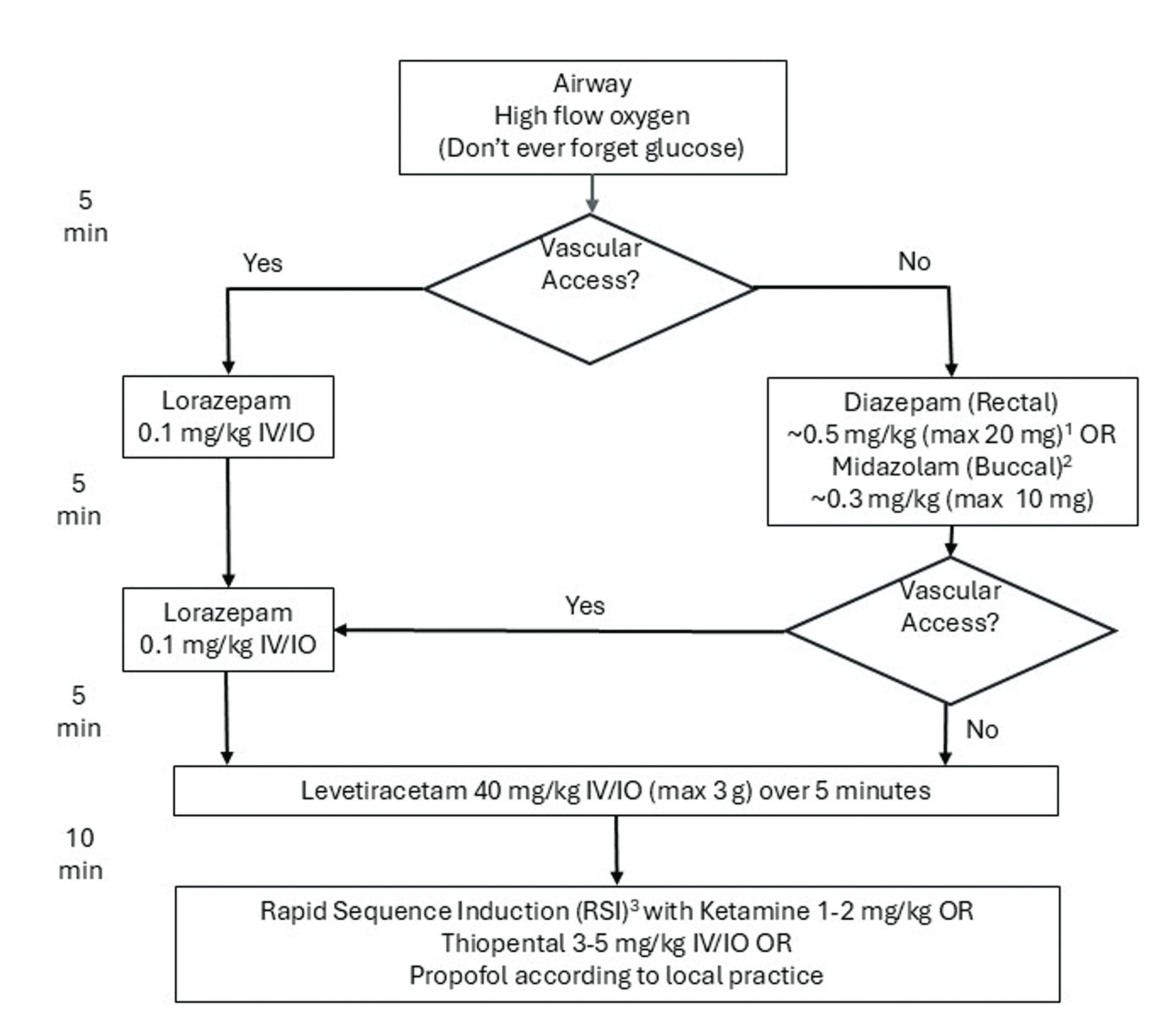
Summary flow chart of the abridged APLS guidelines, 7th edition, 2021 ^1 ^Diazepam (Rectal) dose: 1 month-1year = 5 mg; 2-11 years = 5-10 mg; 12-17 years = 10-20 mg; ^2^ Midazolam (Buccal) dose: 3-11 months = 2.5 mg; 1-4 years = 5 mg; 5-9 years = 7.5 mg; 10-17 years = 10 mg; ^3^ If anaesthetic team not ready for RSI: Phenytoin 20 mg/kg IV/IO (max 2 g) over 20 minutes OR Phenobarbitone 20 mg/kg IV/IO (max 1 g) over 20 minutes, pending RSI APLS: Advanced Paediatric Life Support [26], IV/IO: intravenous or intraosseous; RSI: rapid sequence intubation

The APLS guidelines have evolved since the time of the audit (Figure 2) but still recommend no more than two doses of benzodiazepine: intravenous (IV) or intraosseous (IO), rectal, or buccal, followed by phenytoin or phenobarbitone, or more recently levetiracetam IV/IO [26].

## Results

**Figure 3:**
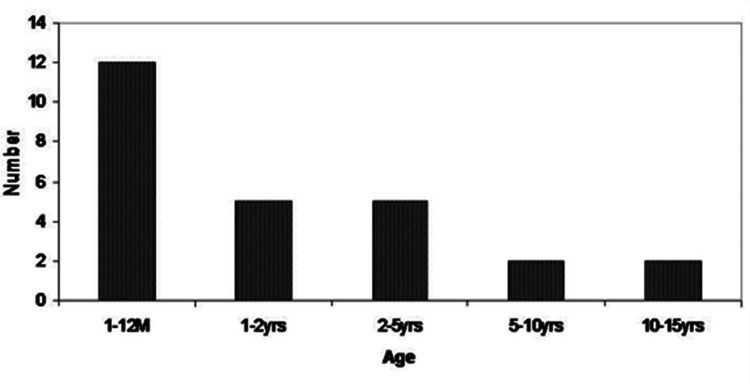
Age distribution (N=26)

Of 739 PICU admissions during the two-year audit period, 36 patients (5%) were admitted for management of epileptic seizures. Four with neonatal seizures were excluded. The remaining 32 had CSE. Records were available for analysis in 26 of the 32 (81%) patients, 16 male, 10 female, median age 14.5 months (range 2.5 months to 13 years). Age distribution was as shown in Figure 3, with 12 (46%) under one year of age.

Of the 26 patients included in the analysis, a previous history of epileptic seizures or epilepsy was present in nine (35%), including two patients with febrile seizures. A previous history of CSE was present in four (15%) patients, including one (4%) with a previous PICU admission for CSE. Twenty-five of the 26 episodes were CSE per se, while one had serial epileptic seizures. Seventeen of 26 (65%) had generalised tonic-clonic seizures, four (15%) had focal, and five (19%) had focal to bilateral tonic-clonic seizures. Prolonged febrile seizure (n=9, 35%) was the most common aetiology (Table 1 and Table 2).

**Table 1:** Aetiology of convulsive status epilepticus

Aetiology	Frequency (Percentage)
Prolonged Febrile Seizure	9 (34)
Acute Symptomatic	7 (27)
Epilepsy related	8 (31)
Idiopathic Status Epilepticus	2 (8)

**Table 2:** Aetiology of acute symptomatic status epilepticus

Aetiology	Frequency (Percentage)
Meningitis	3 (43)
Encephalitis	2 (29)
Non accidental injury	1 (14)
Cerebral infarct	1 (14)

Pre-hospital management

Nineteen of the 26 children (73%) were transferred by ambulance, one by the General Practitioner (GP) in their car, one by parents, and in five children (20%), seizures started while they were inpatients at the hospital. Only six (32%) of the 19 children transferred by ambulance had received pre-hospital treatment. Four patients received one dose of rectal diazepam each, but in three, the administered doses were too low. Two doses of rectal diazepam were given to each of the other two patients, and the doses were too low on both occasions. The child transferred by the GP had been given one appropriate dose of rectal diazepam during transfer. 

The time between the onset of seizure and the administration of the first dose of emergency ASM in children who were transferred from home and who had not received pre-hospital treatment was documented in seven, with a mean of 81 (range 30-265) minutes. There was no record of the time ASMs were administered by ambulance staff or GP, so the time of arrival in the ED was taken as the time by which their pre-hospital dose was administered. This would overestimate the delay in administration. The time from onset of seizure to arrival in the ED was documented in six out of seven children who received pre-hospital treatment, with a mean of 37 (range 17-62) minutes.

**Figure 4:**
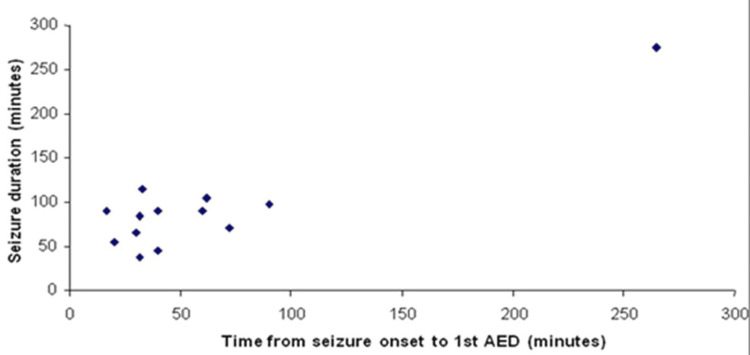
Seizure duration and delay in starting treatment AED: anti-epileptic drug

The relation between the time from seizure onset to first ASM and seizure duration is shown in Figure 4. Pearson product-moment correlation coefficient (r) for the relation between time from seizure onset to first ASM and seizure duration was 0.91, which suggests a statistically significant correlation relationship.

**Figure 5:**
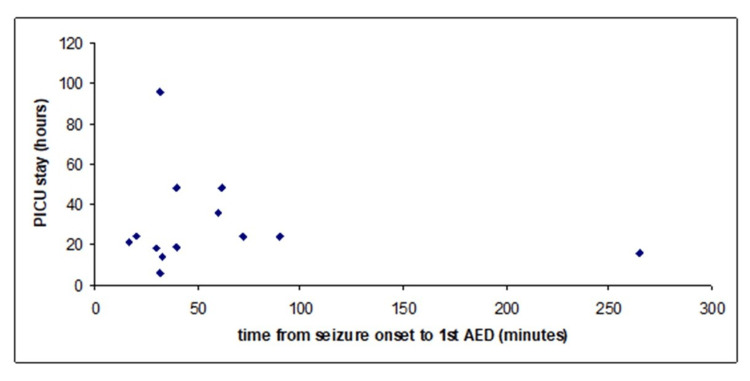
Duration of PICU admission vs delay in start of treatment AED: anti-epileptic drug; PICU: paediatric intensive care unit

The relation between the time from seizure onset to the first ASM and the duration of PICU stay is shown in Figure 5. Pearson product-moment correlation coefficient (r) for the relation between time from seizure onset to first ASM and duration of PICU stay was -0.16, which does not suggest a significant correlation relationship.

The duration of PICU stay seems to be mainly dependent on the aetiology. In children with prolonged febrile seizures, the mean duration of PICU stay was 23 (range 6-48) hours. In those with CSE and established epilepsies, including idiopathic epilepsies, the mean duration of PICU stay was 49 (range 6-144) hours. In those with acute symptomatic CSE, the mean duration of PICU stay was 54 (range 16-168) hours. Those with acute symptomatic CSE without an established epilepsy, and those with established epilepsies, had significantly longer PICU stays (p = 0.045) when compared to those with prolonged febrile seizures.

In children who received pre-hospital treatment, the mean duration of CSE was 90 (range 55-115) minutes, and the mean duration of PICU stay was 38 (range 14-96) hours. In children who had not received pre-hospital treatment, the mean duration of CSE was 177 (range 38-730) minutes, and the mean duration of PICU stay was 47 (range 6-168) hours. These differences were not statistically significant (p = 0.27 and 0.45, respectively). Rescue emergency ASM had not been given by parents/carers to any of these children admitted to PICU.

ED management

The time from seizure onset to arrival at the ED ranged from 17 to 265 (median 34) minutes. Time from arrival to the ED to first emergency ASM ranged from one to 15 (median of 4.5) minutes. All patients had blood glucose measured at the bedside, and 25/26 (96%) received benzodiazepines as first-line medication. Buccal midazolam was given to one patient, although the dose given was too low. The remaining 25 received rectal diazepam in the emergency department.

Seven of 26 (27%) patients received more than two doses of benzodiazepines in total, and in nine (35%), the benzodiazepine doses were too low. Three doses of benzodiazepines were given to four patients, four doses were given to two patients, and six doses were given to one patient. There was no significant difference in duration of ventilation or duration of PICU stay in patients who received one or two doses of benzodiazepines compared to those who received more than two doses. However, five of the 19 (26%) who received one or two doses did not need intubation, whereas all seven (100%) who received more than two doses of benzodiazepines needed intubation and ventilation.

Seventeen patients received a second-line medication, and 13 received a third-line medication. As a second-line medication, rectal paraldehyde was given to 13/17 (76%) children, phenytoin in 3/17 (18%), and phenobarbital (phenobarbitone) in 1/17 (6%). As a third-line medication, phenytoin was given in 5/13 (39%), phenobarbital in 3/13 (23%), rectal paraldehyde in 3/13 (23%), and a benzodiazepine in 2/13 (15%). The doses of all drugs that were given as second- and third-line treatments were appropriate. 

Twenty-one of 26 (81%) patients were intubated and ventilated. The indications for intubation are given in Table 3.

**Table 3:** Indication for Intubation in the paediatric emergency department

Indication	Frequency (Percentage)
Seizure Termination	8 (37%)
Seizure Termination & CT (head)	2 (10%)
Respiratory Depression	4 (19%)
Respiratory Depression & CT (head)	5 (24%)
CT (head)	1 (5%)
Sepsis	1 (5%)

Table 4 shows how APLS guidelines were followed in the management of status epilepticus.

**Table 4:** APLS guideline adherence APLS: Advanced Paediatric Life Support; BZD: benzodiazepine

APLS guideline	Frequency (Percentage)
Strictly followed	10 (38%)
Order followed but the number of BZD doses too many	2 (8%)
Order followed but BZD doses (mg) too low	5 (19%)
Order not followed. Doses (mg) appropriate	4 (16%)
Order not followed. Number of BZD Doses too many	5 (19%)

PICU management

The indications for PICU admission are documented in Table 5.

**Table 5:** Indication for PICU admission PICU: pediatric intensive care unit

Indication	Frequency (Percentage)
Ventilated	20 (76%)
Respiratory Depression	3 (12%)
Continuing Seizures	1 (4%)
Inotropes for Hypotension	1 (4%)
Meningococcal Sepsis	1 (4%)

The duration of ventilation ranged from 0 to 144 (median of 26) hours, and the duration of PICU stay ranged from 6 to 168 (median of 40) hours. Ten patients were transferred in from different district general hospitals. The median duration of ventilation in these 10 patients was 41 (range 0-144) hours, and the median length of PICU stay was 2.5 (range 0.25-7) days. Six of 10 patients (60%) referred from district general hospitals received ventilation for less than 14 hours and were discharged from the PICU within two days.

Twenty-five of 26 (96%) patients survived and were discharged to the wards. One patient with established epilepsy, developmental delay, and an autism spectrum disorder died with multiorgan dysfunction syndrome and severe hypoxic brain injury secondary to prolonged status epilepticus. His MRI scan demonstrated a globally infarcted brain with no blood flow in the intracranial vessels.

## Discussion

This audit was conducted nearly 20 years ago. The audit highlights the paucity of pre-hospital emergency treatment and the poor adherence to the national guidelines. It was presented as an abstract locally and nationally at a time when practice in the United Kingdom was shifting from rectal diazepam to buccal midazolam, to which these findings may have contributed alongside other emerging evidence. The most recent APLS (7th edition) guideline [26] was reviewed, and the use and doses of benzodiazepines in the first two steps are about the same as before, but IV levetiracetam has largely replaced phenytoin in step 3 (Figure 2).

Levetiracetam was as effective or better than phenytoin/fosphenytoin in three well-designed clinical trials published in 2019 [28-30]. It has also been endorsed by the National Institute for Health and Care Excellence (NICE) [25]. It is easier, quicker, and safer to give IV than phenytoin. The use of levetiracetam in place of phenytoin might have prevented some of the children from needing PICU. Another similar clinical audit would be able to assess that.

We believe that it would be helpful to repeat this clinical audit now to see if the updated guidelines and current implementation and training have improved adherence to the guidelines, reduced the proportion of children requiring PICU, and reduced their length of stay. To this end, it was important to publish this audit as a baseline benchmark against which future clinical audits, including those at other hospitals, can be compared. A major learning point from this audit was the frequent use of inappropriate, especially low, doses of benzodiazepines.

CSE in childhood is a life-threatening condition with significant mortality and morbidity. Although the outcome of CSE is mainly determined by its underlying aetiology, the duration of status is also important, so delay in initiation of treatment could result in prolongation of the seizure and increase the morbidity and mortality. The longer the duration of the episode, the more difficult it may be to terminate [9]. Early seizure termination by starting treatment as soon as it seems the seizure may become prolonged is paramount and was reflected in APLS guidelines and their revisions over time. So, it was emphasised in APLS guidelines [21,26] that convulsions that persist beyond five minutes may not stop spontaneously. APLS recommends starting emergency ASM treatment if a generalised or focal becoming bilateral convulsive epileptic seizure (formerly called a secondary generalized seizure) lasts more than five minutes. So, pre-hospital management by paramedical staff or family and carers may be a very important determinant of the outcomes for CSE in children.

Children with longer delays to initial treatment had numerically longer seizures, higher rates of intubation and ventilation, and longer PICU stays, but these differences did not reach statistical significance and should be regarded as hypothesis-generating. Caution is needed in interpreting the relationship between delay in administering emergency treatment and seizure duration, as prolonged seizures could have been treated promptly or after a long delay, while short seizures could only have had prompt treatment or no treatment at all.

Only a third of patients who were transferred by ambulance staff received pre-hospital treatment. Five of those six patients received inappropriately low doses of rectal diazepam. At the time, only rectal or IV diazepam was routinely carried by ambulance crews. Now some carry buccal or IM midazolam as well. We did not want to speculate on whether ambulance crews and paramedics were reluctant to use or lacked confidence in the use of the rectal route in children, or were more concerned about respiratory depression than the epileptic seizure itself, as we have no data to support that. The use of prehospital treatment by ambulance crews would be an important metric in future clinical audits. It would be best if future clinical audits capture the recorded time that ASMs were given pre-hospital by ambulance crews, paramedics, parents, or carers.

There was a significant delay in the initiation of treatment in children who did not receive pre-hospital treatment, with a mean of 81 (range 30-265) minutes. This was unacceptable, and many factors may have contributed, including delay in parents/carers bringing the child to the ED, delay in calling for an ambulance, and travel time to the ED by road. We suspect delays in the administration of the first ASM once in the ED would be only a few minutes, but we do not have that data. This is an important metric to use in future clinical audits.

None of these patients received buccal, nasal, or intramuscular midazolam in pre-hospital settings, although buccal midazolam was in common use amongst patients with epilepsies locally. APLS guidelines suggested giving 0.3-0.5 mg/kg or equivalent of buccal midazolam as an alternative to rectal diazepam. The NICE evidence-based guidelines on the epilepsies [25] also still recommend buccal midazolam as an alternative to rectal diazepam for prolonged or repeated seizures, as did the Scottish Intercollegiate Guideline Network (SIGN) evidence-based guideline on epilepsies in children and young people [24]. A multi-centre randomised controlled trial showed that buccal midazolam was at least as safe as and more effective than rectal diazepam in the ED [16]. Buccal midazolam can be given more easily, is more socially acceptable than rectal diazepam, and is preferred by parents and guardians [15]. It is conceivable that, if available to them, buccal or even intramuscular midazolam would be more readily used by ambulance crews than rectal diazepam, although this clinical audit did not address that question.

There is evidence that giving more than two doses of benzodiazepines increases the risk of respiratory depression, need for ventilation, and PICU admission [9,19,20]. In our study, we noted just over a quarter of patients who needed PICU received more than two doses of benzodiazepines in total, and a quarter of benzodiazepine doses given were inappropriately low. So, care should be taken to avoid excessive as well as inadequate or insufficient doses of benzodiazepines while treating CSE.

Ten patients were transferred to the PICU from different district general hospitals. Six (60%) received ventilation for less than 14 hours and were discharged from the PICU within two days. These figures raise the possibility that short-term paediatric ventilation capability in appropriately resourced district general hospitals might reduce inter-hospital transfers for some children, although this inference is limited by the observational study design. 

Limitations of the study

The number of children included in the clinical audit is small and only represents a single centre, so caution is needed in drawing any generalisable conclusions. Also, the data were not formally tested for normality, so the results of the statistical tests should be viewed with caution. Furthermore, the relationships between time from seizure onset to first ASM and seizure duration, and PICU length of stay are greatly influenced by the outlier with the longest delay in treatment (Figures 4, 5). Short seizures cannot accrue long treatment delays by definition, so there will inevitably be some positive correlation between treatment delay and seizure duration. Given these limitations and the retrospective nature of a clinical audit, the power of this study is insufficient to exclude Type I or Type II errors, and the findings should be viewed as hypothesis-generating only.

However, the study is still useful in hypothesis-generating, rather than demonstrating clear conclusions. The data is now old; however, as demonstrated in Figures 1, 2, the United Kingdom guidelines for early treatment of CSE in children, the core early steps of prompt benzodiazepine administration have remained broadly similar, there have been substantial changes in pre-hospital practice, second-line agent selection, and the near-abandonment of paraldehyde, which complicates the comparability of some treatment metrics. Furthermore, the study highlights a surprising degree of non-adherence to guidelines, which challenges current children's emergency departments to review their own adherence.

## Conclusions

We recommend increasing the awareness among paramedical staff of the importance of giving emergency ASM treatment for CSE promptly, pre-hospital arrival. This might be facilitated by the use of buccal or intramuscular midazolam rather than rectal diazepam. We also recommend continuous education and training of all staff on contemporary guidelines with particular emphasis on timely buccal/IM midazolam administration, avoidance of excessive benzodiazepine doses, and appropriate second-line agent selection, including IV levetiracetam, unless there is a specific reason in any particular child to adopt a different treatment or dose. Retrospective clinical audit of process and outcome against the data reported here is feasible and would help push patient care improvement, both locally, nationally, and further afield.
